# The Association of Blood Urea Nitrogen to Creatinine Ratio and the Prognosis of Critically Ill Patients with Cerebral Infarction: A Cohort Study

**DOI:** 10.1155/2022/2151840

**Published:** 2022-10-10

**Authors:** Ting Chen, Ai-Ping Li, Qi Gong, Lin Zhou, Yi-Xuan Zhao, Zhi-Wen Zhou, Wen-Sheng Zhou

**Affiliations:** Department of Neurology, Hunan Provincial People's Hospital (the First Affiliated Hospital of Hunan Normal University), Changsha 410001, China

## Abstract

**Background:**

To evaluate the association between blood urea nitrogen (BUN) to creatinine (Cr) (BUN/Cr) ratio and the in-hospital mortality of critically ill patients with cerebral infarction in intensive care unit (ICU).

**Methods:**

In this cohort study, the data of 3059 participants with cerebral infarction were collected from the Medical Information Mart for Intensive Care (MIMIC)-III and the MIMIC-IV database. After propensity score matching (PSM) on age and gender, 2085 people were involved in and divided into the alive group (*n* = 1390) and the dead group (*n* = 695) based on the results of follow-up. Multivariate logistic analyses were applied to identify the confounders and the association between BUN/Cr and mortality of cerebral infarction.

**Results:**

The median follow-up time was 10.5 days. Among 2778 participants, 695 were dead at the end of follow-up. Univariate analysis revealed that BUN/Cr [risk ratio (RR) = 1.01, 95% confidence interval (CI): 1.01-1.02] might be associated with the in-hospital mortality of cerebral infarction patients. After adjusting for respiratory failure, malignant cancer, anticoagulation, liver disease, white blood cell (WBC), red cell distribution width (RDW), glucose, bicarbonate, and temperature, BUN/Cr had week correlation with the increased risk of in-hospital mortality of cerebral infarction patients (RR = 1.01, 95% CI: 1.01-1.02).

**Conclusion:**

This study evaluated the association between BUN/Cr and the in-hospital mortality of cerebral infarction patients in ICU and found that BUN/Cr had weak correlation with the increased risk of in-hospital mortality of patients with cerebral infarction in ICU especially in males and those with respiratory failure, malignant cancer, and without liver disease, as well as those receiving anticoagulation.

## 1. Introduction

Cerebral infarction is a kind of brain injury due to the obstruction of blood supply in the brain, which induces ischemic and hypoxic necrosis of innervation [1]. As one of the most common types of cerebrovascular diseases, cerebral infarction accounts for about 70% of all cerebrovascular diseases and approximately 85% of all strokes [2–4]. Cerebral infarction has high incidence, disability, recurrence rate, and mortality, which has resulted in a substantial burden to the society [5]. In China, the reported 1-month morality rate after cerebral infarction was 2.3%-3.2%, and the 3-month mortality rate was 9%-9.6%, and the mortality or disability rate in patients with acute cerebral infarction was 34.5%-37.1% [6]. The estimated mortality of cerebral infarction was about 6.17 million every year all over the world [7]. Nearly 1/3 patients with acute cerebral infarction suffered poor outcomes [8]. To identify essential biomarkers associated with the prognosis of cerebral infarction patients might be helpful for improving the outcomes of these patients.

The fluid metabolism including dehydration status was reported to be potentially associated with the occurrence of cerebral infarction [9]. Dehydration could lead to increased blood viscosity, reduced cardiac output per stroke, impaired collateral blood flow, and decreased cerebral perfusion, which might increase the risk of cerebral infarction [10]. Blood urea nitrogen (BUN) and creatinine (Cr) were metabolic end products of nitrogen-containing substances in human bodies, which were readily available biomarkers of renal function in clinic [11]. BUN to creatinine (BUN/Cr) ratio was a laboratory biomarker frequently used for determining the dehydration [12]. In previous studies, BUN/Cr was identified as an independent prognostic indicator for poor outcomes of patients with different diseases, including stroke [13] and heart failure [14]. Some other studies identified that low level of BUN/Cr might increase the risks of ischemic stroke [15]. The roles of BUN/Cr on diseases were inconformity. Besides, the potential role of BUN/Cr on the prognosis of patients with cerebral infarction was still unclear. Therefore, this study is mainly to evaluate the association between BUN/Cr and the in-hospital mortality of critically ill patients with cerebral infarction in intensive care unit (ICU).

In the present study, we evaluated the association between BUN/Cr and the in-hospital mortality of critically ill patients with cerebral infarction in intensive care unit (ICU) based on the data from the Medical Information Mart for Intensive Care (MIMIC)-III and the MIMIC-IV. We also stratified the analysis on age, gender, and whether the patient was complicated with respiratory failure, malignant cancers, or liver diseases or whether the patient received anticoagulation treatments.

## 2. Materials and Methods

### 2.1. Study Design and Population

In this cohort study, the data of 3059 participants with cerebral infarction were collected from MIMIC database, including 1568 in the MIMIC-III and 1491 in the MIMIC-IV. The MIMIC-III is a large, single-center open database comprising the electronic health records including demographic characteristics, monitoring vital signs, laboratory and microbiological examination, imaging examination, observation and recording of intake and output, drug treatment, length of stay, survival data, and discharge or death records of more than 60,000 individuals admitted to an ICU at the Beth Israel Deaconess Medical Center between 2001 and 2012 [16]. The MIMIC-IV database is an updated version of the MIMIC-III, and improvements have been made including simplifying the structure, adding new data elements, and improving the usability of previous data elements. The MIMIC-IV involves the comprehensive and high-quality electronic health records of patients admitted to the ICU or emergency department of the Beth Israel Deaconess Medical Center from 2008 to 2019 [17]. The database got the approval from the institutional review boards of the Massachusetts Institute of Technology (Cambridge, Massachusetts) and the Beth Israel Deaconess Medical Center (Boston, Massachusetts). Cerebral infarction in patients was diagnosed when admitted to ICUs based on International Classification of Diseases, Ninth Revision (ICD-9) code and the Tenth Revision (ICD-10) code. ICD-9: 43301, 43311, 43321, 43331, 43381, 43391, 43401, 43411, and 43491; ICD-10: I63. In our study, patients who aged <18 years were excluded. Those admitted to ICU <24 h or who had no data on BUN/Cr were also excluded. Finally, the data of 2778 participants were analyzed. At the end of the follow-up, 2083 patients were alive, and 695 patients were dead. After propensity score matching (PSM) on age and gender, 2085 people were involved in and divided into the alive group (*n* = 1390) and the dead group (*n* = 695).

### 2.2. Variables

The main variable investigated was BUN/Cr. Covariables analyzed in the study included comorbidities (congestive heart failure (CHF) (yes or no), atrial fibrillation (AF) (yes or no), diabetes mellitus (yes or no), respiratory failure (yes or no), renal failure (yes or no), malignant cancer (yes or no), hypertension (yes or no), and liver disease (yes or no)), medication use (thrombolytic (yes or no) and anticoagulation (yes or no)), laboratory data (heart rate (time/min), systolic blood pressure (SBP) (mmhg), diastolic blood pressure (DBP) (mmhg), mean arterial pressure (MAP) (mmhg), respiratory rate (time/min), temperature (°C), white blood cell (WBC) (K/*μ*l), platelets (PLT) (K/*μ*l), hemoglobin (g/dL), red cell distribution width (RDW) percent, hematocrit percent, Cr (mg/dl), International Normalized Ratio (INR), BUN (mg/dl), glucose (mg/dl), bicarbonate (mEq/l), sodium (mEq/l), and potassium (mEq/l)), and the Sequential Organ Failure Assessment (SOFA) Score, the Simplified Acute Physiology Score II (SAPSII), the Oxford Acute Severity of Illness Score (OASIS), and Charlson comorbidity index.

### 2.3. Outcome Variable

The outcome in the present study was the in-hospital death of participants with cerebral infarction in ICU. All subjects in ICU were followed up until death or discharge. The median follow-up time was 10.5 days. Among 2778 participants, 695 were dead at the end of follow-up.

### 2.4. Sensitivity Analysis

The missing values of all variables were shown in Supplementary Table 1. The results of sensitivity analysis revealed that no statistical difference was observed in the data before and after multi-interpolation. As exhibited in Supplementary Table 2, the age was statistically different between the alive group and the dead group (66.42 years vs. 71.51 years). To make the baseline data equilibrated between the alive group and the dead group, PSM was applied. After PSM, the data of age and gender showed no statistical difference between the two groups.

### 2.5. Statistical Analysis

The continuous variables were presented in the forms of mean ± standard deviation (SD) if the data were normally distributed or M (Q_1_, Q_3_) if the data were not normally distributed. Student's *t*-test was used to compare the difference between groups. The categorical variables were displayed as *n* (%), and chi-square and Wilcoxon rank sum test were applied to judge the differences between groups. The data with missing value <10% were multi-interpolated and with missing value ≥10% were excluded. PSM was performed on the data. Sensitivity analysis was performed between the data before multi-interpolation and after multi-interpolation as well as before PSM and after PSM. Multivariate logistic analyses were applied to identify the confounders of the association between BUN/Cr and mortality of cerebral infarction. Model 1 included all variables with statistical difference between the alive group and the dead group. Model 2 included BUN/Cr, respiratory failure, malignant cancer, anticoagulation, liver disease, temperature, WBC, RDW percent, glucose, and bicarbonate. Subgroup analysis was performed to assess the association between BUN/Cr and the mortality of cerebral infarction in different groups of people concerning age, gender, and whether the patient was complicated with respiratory failure, malignant cancer, or liver disease or whether the patient received anticoagulation treatments. The risk ratio (RR) was employed to evaluate the association between BUN/Cr and the mortality of cerebral infarction in ICU. The confidence level was set as *α* = 0.05. All statistical analysis was conducted via SAS 9.4, and R 4.0.3.

## 3. Results

### 3.1. Comparisons of the Baseline Characteristics between the Alive Group and the Dead Group

**Figure 1:**
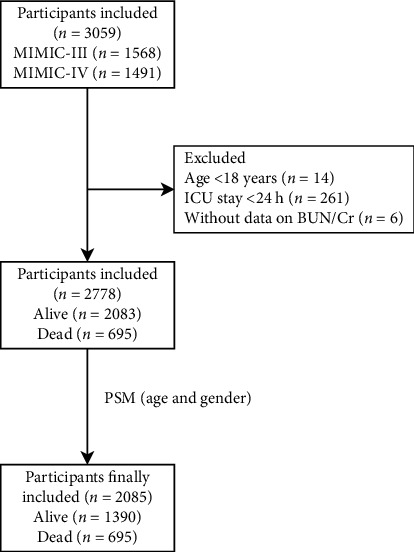
The screen process of participants in this study.

This study was a cohort study involving the data of 3059 participants with cerebral infarction. Among all participants, patients aged <18 years (*n* = 14) and those admitted to ICU <24 h (*n* = 261) were excluded. Also, 6 patients without data on BUN/Cr were excluded. Finally, the data of 2778 participants were analyzed. After PSM on age and gender, 2085 people were finally included in our study and divided into the alive group (*n* = 1390) and the dead group (*n* = 695). The detailed screen process was displayed in Figure 1.

As observed in Table 1, the percentages of patients with CHF (26.12% vs. 30.79%), respiratory failure (31.08% vs. 53.67%), renal failure (19.78% vs. 26.91%), malignant cancer (11.94% vs. 18.27%), and liver disease (4.96% vs. 10.65%) were lower in the alive group than the dead group. The percentage of patients with anticoagulation use in the alive group was higher than the dead group (90.36% vs. 85.47%). The average heart rate (83.56 times/min vs. 88.95 times/min), RDW (14.37% vs. 15.01%), potassium level (4.15 mEq/l vs. 4.24 mEq/l), and OASIS (31.53 vs. 36.17) in the alive group were lower than the dead group. The average temperature (36.59°C vs. 36.26°C), hematocrit (12.03 g/dl vs. 11.55 g/dl), hematocrit (36.05% vs. 34.93%), and bicarbonate (23.91 mEq/l vs. 23.00 mEq/l) were higher in the alive group than in the dead group. The median SOFA score (2 vs. 4), SAPSII (35 vs. 43), Cr (1.00 mg/dl vs. 1.10 mg/dl), BUN (19 mg/dl vs. 22 mg/dl), and BUN/Cr (19.00 vs. 20.00) were lower in the alive group than in the dead group.

### 3.2. The Association between BUN/Cr and Mortality of Participants with Cerebral Infarction

The variables with statistical difference between the alive group and the dead group were included in the multivariable regression analysis model 1. The results depicted that BUN/Cr (RR = 1.01, 95% CI: 1.01-1.02), respiratory failure (RR = 2.34, 95% CI: 1.90-2.87), malignant cancer (RR = 1.68, 95% CI: 1.27-2.22), anticoagulation (RR = 0.44, 95% CI: 0.33-0.60), liver disease (RR = 1.63, 95% CI: 1.11-2.37), WBC (RR = 1.01, 95% CI: 1.00-1.03), RDW percent (RR = 1.08, 95% CI: 1.03-1.14), glucose (RR = 1.01, 95% CI: 1.01-1.01), bicarbonate (RR = 0.97, 95% CI: 0.95-0.99), and temperature (RR = 0.95, 95% CI: 0.92-0.99) might be associated with the in-hospital mortality of cerebral infarction patients. After adjusting for respiratory failure, malignant cancer, anticoagulation, liver disease, WBC, RDW, glucose, bicarbonate, and temperature, BUN/Cr was associated with increased risk of in-hospital mortality of cerebral infarction patients (RR = 1.01, 95% CI: 1.01-1.02) (Table 2).

### 3.3. The Association between BUN/Cr and Mortality of Patients with Cerebral Infarction in Different Subgroups

According to the data in Table 3, no significant effect of BUN/Cr on the in-hospital mortality was observed in patients aged ≥65 years or <65 years (all *P* > 0.05). In terms of gender, BUN/Cr was associated 1.02-fold risk of in-hospital mortality in male patients with cerebral infarction in ICU (RR = 1.02, 95% CI: 1.01-1.04), but had no statistical effect on female patients (*P* > 0.05). BUN/Cr was correlated with the increased risk of in-hospital mortality in cerebral infarction patients with respiratory failure (RR = 1.02, 95% CI: 1.01-1.03) or malignant cancer (RR = 1.03, 95% CI: 1.01-1.05). In cerebral infarction patients that received anticoagulation, the risk of in-hospital morality was increased by 0.01 as one-unit increase of BUN/Cr (RR = 1.01, 95% CI: 1.01-1.02). As in patients without liver disease, BUN/Cr was a risk factor for the in-hospital mortality of cerebral infarction patients in ICU (RR = 1.01, 95% CI: 1.01-1.02).

## 4. Discussion

In the current study, the effect of BUN/Cr on the in-hospital mortality of cerebral infarction patients in ICU was assessed. The results delineated that BUN/Cr had weak correlation with the increased risk of in-hospital mortality of patients with cerebral infarction in ICU. In addition, the increased risk of in-hospital mortality of cerebral infarction patients was also observed in males and those with respiratory failure, malignant cancer, and without liver disease, as well as those receiving anticoagulation. The findings of our study might highlight the role of BUN/Cr in the prognosis of patients with cerebral infarction, remind the clinicians to be concerned about the BUN/Cr level of patients, and provide some interventions for those with a high level of BUN/Cr.

Herein, we found that BUN/Cr had weak association with the increased risk of in-hospital mortality of patients with cerebral infarction in ICU. BUN/Cr was a composite index of BUN and Cr, and the added value in addition to BUN or Cr was explored before. In a study of Akimoto et al., BUN/Cr was disproportionately increased in patients with cerebral infarction, which might be the contributor for the occurrence of cerebral infarction [18]. Previously, Kim et al. depicted that BUN/Cr was an independent risk factor for venous thromboembolism in patients with acute ischemic stroke [19]. In addition, the increased BUN/Cr ratio was associated with poor outcomes in patients with ischemic stroke [20]. BUN/Cr ≥15 was reported to be an independent risk factor for predicting the long-term outcome of thrombolyzed patients with acute ischemic stroke [21]. The mechanisms underlying the association between BUN/Cr and the in-hospital mortality of cerebral infarction in ICU might be due to that BUN/Cr was an important indicator for dehydration, which was indicated by an increased BUN/Cr ratio and served a clinical maker for deterioration in acute stroke [22]. Dehydration could reduce the brain perfusion and impair the neuroplasticity [23, 24]. Previous studies also indicated that dehydration might increase the event of venous thromboembolism after acute infarction [25]. In addition, BUN/Cr levels were also associated with abnormal inflammation [26], oxidative stress [27], and endothelial dysfunction [28] in patients, which might be other potential mechanisms.

In this study, the association between BUN/Cr and the increased risk of in-hospital mortality of cerebral infarction patients was observed in subgroups including males and those with respiratory failure, malignant cancer, and without liver disease, as well as those receiving anticoagulation. Patients with respiratory failure and malignant cancer might be associated with a higher infection and inflammation status [29, 30]. Anticoagulation was applied for the treatment of venous thrombosis and prophylaxis of postoperative venous thrombosis [31]. Anticoagulation was reported to be an influencing factor of hemorrhagic transformation in nonthrombolysis patients with cerebral infarction [32]. In patients receiving the anticoagulation, abnormal BUN/Cr ratio might be a more reliable indicator associated with the mortality in cerebral infarction patients. For male patients and those with respiratory failure, malignant cancer, and without liver disease, as well as those receiving anticoagulation, BUN/Cr level should be paid some attention. For those with high BUN/Cr levels, appropriate interventions should be provided.

Our study evaluated the role of BUN/Cr on the in-hospital mortality of cerebral infarction patients in ICU. BUN/Cr was identified as an indicator for in-hospital mortality of these patients. BUN and Cr are easily obtained biomarkers and can be widely applied in clinic. We also performed subgroup analyses and found the associations of BUN/Cr with specific cerebral infarction patients. The limitation in the present study was that the data were extracted from MIMIC-III and MIMIC-IV database, and some important variables including the detailed data on the treatments during ICU stay were not included, which might affect the outcomes of patients. In the future, more randomized controlled trials were required to verify the findings of the current study.

## 5. Conclusion

This study evaluated the association between BUN/Cr and the in-hospital mortality of cerebral infarction patients in ICU and found that BUN/Cr had weak correlation with the increased risk of in-hospital mortality of patients with cerebral infarction in ICU especially in males and those with respiratory failure, malignant cancer, and without liver disease, as well as those receiving anticoagulation. The findings of our study reminded the clinicians to pay attention on the level of BUN/Cr in cerebral infarction patients in ICU.

## Figures and Tables

**Table 1:** Comparisons of the baseline characteristics between the alive group and the dead group.

Variables	Total (*n* = 2085)	Alive (*n* = 1390)	Dead (*n* = 695)	Statistics	*P*
CHF, *n* (%)				*χ* ^2^ = 5.062	0.024
No	1508 (72.33)	1027 (73.88)	481 (69.21)		
Yes	577 (27.67)	363 (26.12)	214 (30.79)		
AF, *n* (%)				*χ* ^2^ = 0.164	0.686
No	1154 (55.35)	765 (55.04)	389 (55.97)		
Yes	931 (44.65)	625 (44.96)	306 (44.03)		
Diabetes mellitus, *n* (%)				*χ* ^2^ = 0.192	0.662
No	1451 (69.59)	963 (69.28)	488 (70.22)		
Yes	634 (30.41)	427 (30.72)	207 (29.78)		
Respiratory failure, *n* (%)				*χ* ^2^ = 99.754	<.001
No	1280 (61.39)	958 (68.92)	322 (46.33)		
Yes	805 (38.61)	432 (31.08)	373 (53.67)		
Renal failure, *n* (%)				*χ* ^2^ = 13.627	<.001
No	1623 (77.84)	1115 (80.22)	508 (73.09)		
Yes	462 (22.16)	275 (19.78)	187 (26.91)		
Malignant cancer, *n* (%)				*χ* ^2^ = 15.376	<.001
No	1792 (85.95)	1224 (88.06)	568 (81.73)		
Yes	293 (14.05)	166 (11.94)	127 (18.27)		
Thrombolytic, *n* (%)				*χ* ^2^ = 3.202	0.074
No	1777 (85.23)	1171 (84.24)	606 (87.19)		
Yes	308 (14.77)	219 (15.76)	89 (12.81)		
Anticoagulation, *n* (%)				*χ* ^2^ = 11.088	<.001
No	235 (11.27)	134 (9.64)	101 (14.53)		
Yes	1850 (88.73)	1256 (90.36)	594 (85.47)		
Hypertension, *n* (%)				*χ* ^2^ = 3.542	0.060
No	793 (38.03)	509 (36.62)	284 (40.86)		
Yes	1292 (61.97)	881 (63.38)	411 (59.14)		
Liver disease, *n* (%)				*χ* ^2^ = 23.429	<.001
No	1942 (93.14)	1321 (95.04)	621 (89.35)		
Yes	143 (6.86)	69 (4.96)	74 (10.65)		
Heart rate (time/min), mean ± SD	85.36 ± 20.10	83.56 ± 18.71	88.95 ± 22.21	*t* = −5.50	<.001
SBP (mmhg), M(Q_1_,Q_3_)	137 (116, 155)	138 (118,155)	134 (113,155)	*Z* = −2.151	0.031
DBP (mmhg), M (Q_1_, Q_3_)	70 (58, 83)	70 (58, 83)	70 (58, 82)	*Z* = −0.187	0.851
MAP (mmhg), M (Q_1_, Q_3_)	89 (76, 102)	89 (77, 103)	88 (75, 102)	*Z* = −1.072	0.284
Respiratory rate (time/min), M (Q_1_, Q_3_)	18.00 (15.00, 22.00)	18.00 (15.00, 21.00)	19.00 (15.00, 24.00)	*Z* = 4.577	<.001
Temperature (°C), mean ± SD	36.48 ± 2.72	36.59 ± 1.80	36.26 ± 3.96	*t* = 2.15	0.032
SOFA, M (Q_1_, Q_3_)	2.00 (1.00, 5.00)	2.00 (0.00, 4.00)	4.00 (1.00, 6.00)	*Z* = 7.945	<.001
SAPSII, M (Q_1_, Q_3_)	38.00 (30.00, 47.00)	35.00 (28.00, 44.00)	43.00 (35.00, 53.00)	*Z* = 12.891	<.001
OASIS, mean ± SD	33.08 ± 9.92	31.53 ± 9.68	36.17 ± 9.67	*t* = −10.33	<.001
WBC (K/*μ*l), M(Q_1_, Q_3_)	10.30 (7.80, 13.80)	9.90 (7.70, 13.30)	11.50 (8.00, 15.80)	*Z* = 5.198	<.001
PLT (K/*μ*l), M (Q_1_, Q_3_)	215.00 (160.00, 279.00)	217.00 (164.00, 276.00)	211.00 (153.00, 285.00)	*Z* = −1.257	0.209
Hemoglobin (g/dl), mean ± SD	11.87 ± 2.31	12.03 ± 2.29	11.55 ± 2.32	*t* = 4.55	<.001
RDW percent, mean ± SD	14.59 ± 1.99	14.37 ± 1.88	15.01 ± 2.11	*t* = −6.79	<.001
Hematocrit percent, mean ± SD	35.68 ± 6.51	36.05 ± 6.48	34.93 ± 6.53	*t* = 3.70	<.001
Creatinine (mg/dl), M (Q_1_,Q_3_)	1.00 (0.80, 1.40)	1.00 (0.80, 1.30)	1.10 (0.80, 1.60)	*Z* = 4.686	<.001
INR, M (Q_1_,Q_3_)	1.20 (1.10, 1.40)	1.20 (1.10, 1.30)	1.20 (1.10, 1.40)	*Z* = 6.144	<.001
BUN (mg/dl), M (Q_1_,Q_3_)	20.00 (14.00, 29.00)	19.00 (14.00, 27.00)	22.00 (16.00, 34.00)	*Z* = 6.401	<.001rep
Glucose (mg/dl), M (Q_1_,Q_3_)	130.00 (107.00, 167.00)	126.00 (106.00, 160.00)	136.00 (110.00, 184.00)	*Z* = 4.853	<.001
Bicarbonate (mEq/l), mean ± SD	23.61 ± 4.26	23.91 ± 3.97	23.00 ± 4.72	*t* = 4.36	<.001
Sodium (mEq/l), mean ± SD	139.24 ± 4.64	139.37 ± 4.42	138.98 ± 5.03	*t* = 1.77	0.076
Potassium (mEq/l), mean ± SD	4.18 ± 0.77	4.15 ± 0.73	4.24 ± 0.83	*t* = −2.49	0.013
Charlson comorbidity index, M (Q_1_,Q_3_)	5.00 (3.00, 8.00)	5.00 (3.00, 7.00)	6.00 (3.00, 8.00)	*Z* = 2.891	0.004
BUN/Cr, M (Q_1_, Q_3_)	19.38 (15.00, 24.44)	19.00 (15.00, 23.75)	20.00 (15.00, 25.71)	*Z* = 2.756	0.006

CHF: congestive heart failure; AF: atrial fibrillation; SBP: systolic blood pressure; DBP: diastolic blood pressure; MAP: mean arterial pressure; WBC: white blood cell; PLT: platelets; RDW: red cell distribution width; INR: International Normalized Ratio; SOFA: the Sequential Organ Failure Assessment; SAPSII: the Simplified Acute Physiology Score II; OASIS: the Oxford Acute Severity of Illness Score; BUN: blood urea nitrogen; Cr: creatinine.

**Table 2:** The association between BUN/Cr and mortality of participants with cerebral infarction.

Variables	Model 1		Model 2	
	RR (95% CI)	*P*	RR (95% CI)	*P*
BUN/CR	1.01 (1.01-1.02)	0.045	1.01 (1.01-1.02)	0.025
CHF	0.98 (0.78-1.24)	0.884		
Respiratory failure	2.34 (1.90-2.87)	<0.001	2.50 (2.04-3.05)	<0.001
Renal failure	1.10 (0.86-1.41)	0.438		
Malignant cancer	1.68 (1.27-2.22)	<0.001	1.72 (1.31-2.26)	<0.001
Anticoagulation	0.44 (0.33-0.60)	<0.001	0.46 (0.34-0.61)	<0.001
Liver disease	1.63 (1.11-2.37)	0.012	1.72 (1.19-2.49)	0.004
Heart rate	1.00 (1.00-1.01)	0.086		
SBP	1.00 (1.00-1.01)	0.259		
Respiratory rate	1.02 (1.00-1.03)	0.088		
Temperature	0.95 (0.92-0.99)	0.007	0.95 (0.93-0.97)	<0.001
WBC	1.01 (1.00-1.03)	0.023	1.01 (1.01-1.03)	0.023
Hemoglobin	0.91 (0.76-1.08)	0.270		
RDW percent	1.08 (1.03-1.14)	0.004	1.11 (1.06-1.16)	<0.001
Hematocrit percent	1.03 (0.97-1.10)	0.281		
INR	0.98 (0.89-1.09)	0.740		
Albumin	0.94 (0.79-1.11)	0.451		
Glucose	1.01 (1.01-1.01)	0.008	1.01 (1.01-1.01)	0.002
Bicarbonate	0.97 (0.95-0.99)	0.033	0.96 (0.94-0.99)	0.001
Sodium	0.99 (0.98-1.00)	0.248		
Potassium	1.05 (0.93-1.19)	0.402		
Charlson comorbidity index	1.01 (0.98-1.05)	0.552		

Mode l included variables with statistical difference between the alive group and the dead group. Model 2: multivariable logistic regression analysis adjusting respiratory failure, malignant cancer, anticoagulation, liver disease, temperature, WBC, RDW percent, glucose, and bicarbonate. CHF: congestive heart failure; SBP: systolic blood pressure; WBC: white blood cell; RDW: red cell distribution width; INR: International Normalized Ratio; BUN: blood urea nitrogen; Cr: creatinine.

**Table 3:** The association between BUN/Cr and mortality of patients with cerebral infarction in different subgroups.

Subgroup	Model	
	RR (95% CI)	*P*
Age		
≥65	1.01 (1.00-1.02)	0.099
<65	1.01 (0.99-1.03)	0.273
Gender		
Male	1.02 (1.01-1.04)	0.023
Female	1.01 (0.99-1.02)	0.445
Respiratory failure		
Yes	1.02 (1.01-1.03)	0.041
No	1.01 (0.99-1.03)	0.286
Malignant cancer		
Yes	1.03 (1.01-1.05)	0.032
No	1.01 (1.00-1.02)	0.126
Anticoagulation		
Yes	1.01 (1.01-1.02)	0.028
No	1.02 (0.98-1.05)	0.341
Liver disease		
Yes	1.02 (0.99-1.05)	0.242
No	1.01 (1.01-1.02)	0.040

Multivariable logistic regression analysis adjusting respiratory failure, malignant cancer, anticoagulation, liver disease, temperature, WBC, RDW percent, glucose, and bicarbonate; BUN: blood urea nitrogen; Cr: creatinine.

## Data Availability

The datasets used and/or analyzed during the current study are available from the corresponding author on reasonable request.

## Abbreviations

MIMIC: The Medical Information Mart for Intensive Care
ICU: Intensive care unit
RR: Risk ratio
CI: Confidence interval
CHF: Congestive heart failure
AF: Atrial fibrillation
SBP: Systolic blood pressure
DBP: Diastolic blood pressure
MAP: Mean arterial pressure
WBC: White blood cell
PLT: Platelets
RDW: Red cell distribution width
INR: International Normalized Ratio
SOFA: The Sequential Organ Failure Assessment
SAPSII: The Simplified Acute Physiology Score II
OASIS: The Oxford Acute Severity of Illness Score
BUN: Blood urea nitrogen
Cr: Creatinine
ICD-9: International Classification of Diseases, Ninth Revision (ICD-9).

## Supplementary Materials

Supplementary Materials Supplementary Table 1: the missing values of all variables and sensitivity analysis before and after manipulation. CHF: congestive heart failure; AF: atrial fibrillation; SBP: systolic blood pressure; DBP: diastolic blood pressure; MAP: mean arterial pressure; WBC: white blood cell; PLT: platelets; RDW: red cell distribution width; INR: International Normalized Ratio; SOFA: the Sequential Organ Failure Assessment; SAPSII: the Simplified Acute Physiology Score II; OASIS: the Oxford Acute Severity of Illness Score; BUN: blood urea nitrogen; Cr: creatinine. Supplementary Table 2: the data before and after PSM. PSM: propensity score matching. The original data has been uploaded to http://figshare.com. doi:10.6084/m9.figshare.19518868
