# Supplementary material for: The Association of Blood Urea Nitrogen to Creatinine Ratio and the Prognosis of Critically Ill Patients with Cerebral Infarction: A Cohort Study
Source: Mediators Inflamm. 2022 Oct 10;2022:2151840. doi: 10.1155/2022/2151840 (PMC9576422; doi:10.1155/2022/2151840)
Supplement: Supplementary Materials — Supplementary Table 1: the missing values of all variables and sensitivity analysis before and after manipulation. CHF: congestive heart failure; AF: atrial fibrillation; SBP: systolic blood pressure; DBP: diastolic blood pressure; MAP: mean arterial pressure; WBC: white blood cell; PLT: platelets; RDW: red cell distribution width; INR: International Normalized Ratio; SOFA: the Sequential Organ Failure Assessment; SAPSII: the Simplified Acute Physiology Score II; OASIS: the Oxford Acute Severity of Illness Score; BUN: blood urea nitrogen; Cr: creatinine. Supplementary Table 2: the data before and after PSM. PSM: propensity score matching. The original data has been uploaded to http://figshare.com. doi:10.6084/m9.figshare.19518868 [file 2151840.f1.docx]

Supplementary Table 1 The missing values of all variables and sensitivity analysis before and after manipulation

| Variable | Miss, n (%) | After | Before | Statistical magnitude | *P* |
| --- | --- | --- | --- | --- | --- |
| Age | 0 (0.00) |  |  |  |  |
| Gender | 0 (0.00) |  |  |  |  |
| CHF | 0 (0.00) |  |  |  |  |
| AF | 0 (0.00) |  |  |  |  |
| Diabetes mellitus | 0 (0.00) |  |  |  |  |
| Respiratory failure | 0 (0.00) |  |  |  |  |
| Renal failure | 0 (0.00) |  |  |  |  |
| Malignant cancer | 0 (0.00) |  |  |  |  |
| Thrombolytic | 0 (0.00) |  |  |  |  |
| Anticoagulation | 0 (0.00) |  |  |  |  |
| Hypertension | 0 (0.00) |  |  |  |  |
| Liver disease | 0 (0.00) |  |  |  |  |
| Heart Rate | 6 (0.22) | 85.11 ± 19.98 | 85.13 ± 20.00 | t=-0.03 | 0.977 |
| SBP | 6 (0.22) | 136 (116, 154) | 136 (116, 154) | Z=0.015 | 0.988 |
| DBP | 6 (0.22) | 70 (58, 82) | 70 (58, 82.5) | Z=0.066 | 0.947 |
| MAP | 6 (0.22) | 89 (77, 102) | 89 (77, 102) | Z=0.014 | 0.989 |
| Respiratory rate | 6 (0.22) | 18.87 ± 6.09 | 18.88 ± 6.10 | t=-0.04 | 0.971 |
| Temperature | 9 (0.32) | 36.46 ± 2.92 | 36.46 ± 2.92 | t=0.02 | 0.988 |
| SOFA | 0 (0.00) |  |  |  |  |
| SAPSII | 0 (0.00) |  |  |  |  |
| OASIS | 0 (0.00) |  |  |  |  |
| WBC | 0 (0.00) |  |  |  |  |
| PLT | 0 (0.00) |  |  |  |  |
| Hemoglobin | 0 (0.00) |  |  |  |  |
| RDW percent | 0 (0.00) |  |  |  |  |
| Hematocrit percent | 0 (0.00) |  |  |  |  |
| Creatinine | 0 (0.00) |  |  |  |  |
| INR | 32 (1.15) | 1.20 (1.10, 1.30) | 1.20 (1.10, 1.30) | Z=0.278 | 0.781 |
| BUN | 0 (0.00) |  |  |  |  |
| Glucose | 0 (0.00) |  |  |  |  |
| Bicarbonate | 0 (0.00) |  |  |  |  |
| Sodium | 0 (0.00) |  |  |  |  |
| Potassium | 0 (0.00) |  |  |  |  |
| Charlson comorbidity index | 0 (0.00) |  |  |  |  |
| BUN/Cr | 0 (0.00) |  |  |  |  |

CHF: congestive heart failure, AF: atrial fibrillation, SBP: systolic blood pressure, DBP: diastolic blood pressure, MAP: mean arterial pressure, WBC: white blood cell, PLT: platelets, RDW: red cell distribution width, INR: International Normalized Ratio, SOFA: the Sequential Organ Failure Assessment, SAPSII: the Simplified Acute Physiology Score II, OASIS: the Oxford Acute Severity of Illness Score, BUN: blood urea nitrogen, Cr: creatinine

Supplementary Table 2 The data before and after PSM

| Variables |  | Before | | | |  | After | | | | |  |
| --- | --- | --- | --- | --- | --- | --- | --- | --- | --- | --- | --- | --- |
|  |  | Alive (n=2083) | Dead (n=695) |  | *P* | | |  | Alive (n=1390) | Dead (n=695) | *P* | |
| Age, Mean±SD |  | 66.42 ± 15.90 | 71.51 ± 14.74 |  | **<.001** | | |  | 71.34 ± 14.50 | 71.51 ± 14.74 | 0.804 | |
| Gender, n (%) |  |  |  |  | 0.076 | | |  |  |  | 0.495 | |
| Female |  | 1010 (48.49) | 364 (52.37) |  |  | | |  | 706 (50.79) | 364 (52.37) |  | |
| Male |  | 1073 (51.51) | 331 (47.63) |  |  | | |  | 684 (49.21) | 331 (47.63) |  | |

PSM: propensity score matching
